# Cardiac-to-adipose axis in metabolic homeostasis and diseases: special instructions from the heart

**DOI:** 10.1186/s13578-023-01097-1

**Published:** 2023-09-04

**Authors:** Songling Tang, Ruixin Li, Wen Ma, Liu Lian, Jiuyu Gao, Yu Cao, Lu Gan

**Affiliations:** 1 Department of Emergency Medicine and Laboratory of Emergency Medicine, West China Hospital, West China School of Medicine, Sichuan University Chengdu, Chengdu, 610041 People’s Republic of China; 2 Sichuan University-The Hong Kong Polytechnic University Institute for Disaster Management and Reconstruction, Chengdu, China

## Abstract

Adipose tissue is essential for maintaining systemic metabolic homeostasis through traditional metabolic regulation, endocrine crosstalk, and extracellular vesicle production. Adipose dysfunction is a risk factor for cardiovascular diseases. The heart is a traditional pump organ. However, it has recently been recognized to coordinate interorgan cross-talk by providing peripheral signals known as cardiokines. These molecules include specific peptides, proteins, microRNAs and novel extracellular vesicle-carried cargoes. Current studies have shown that generalized cardiokine-mediated adipose regulation affects systemic metabolism. Cardiokines regulate lipolysis, adipogenesis, energy expenditure, thermogenesis during cold exposure and adipokine production. Moreover, cardiokines participate in pathological processes such as obesity, diabetes and ischemic heart injury. The underlying mechanisms of the cardiac-to-adipose axis mediated by cardiokines will be further discussed to provide potential therapeutic targets for metabolic diseases and support a new perspective on the need to correct adipose dysfunction after ischemic heart injury.

## Introduction

Adipose tissue, which is the most vital organ that regulates and coordinates systemic metabolism [1], is responsible for lipid storage, energy homeostasis, and whole-body insulin sensitivity [2]. Recent evidence has shown that various remote organs are involved in regulating adipose function in endocrine manners, including weight gain [3], fat mass, adipocyte size, lipid metabolism (lipolysis [4] and lipogenesis) and glucose metabolism [3] (glucose level control and insulin sensitivity), adipogenesis [5, 6], adipose inflammation [3] and adipokine biosynthesis [7, 8]. There are currently many secretory components with endocrine regulatory effects, including traditional cytokines and secretory peptides [5], as well as recently discovered extracellular vesicles and their cargoes [9].

The heart is a conventional pump organ that continuously supplies blood to other tissues. Since the discovery of atrial natriuretic peptide (ANP), the heart has been redefined as an endocrine organ that regulates the functions of other organs or tissues by producing and releasing specialized molecules into circulation. In addition to ANP, cardiac tissues also secrete atrial natriuretic factor (ANF), brain natriuretic peptide (BNP), follistatin like (FSTL) 1, angiotensin II (Ang II) and tumor necrosis factor-α (TNF-α), which are known as cardiokines, emphasizing their source. Cardiokines participate in cell growth and death, myocardial hypertrophy, fibrosis and remodeling through autocrine or paracrine regulation [10, 11], such as bidirectional endothelial-myocardial sympathetic interactions [12]. However, the communication mediated by cardiokines between the heart and peripheral tissues remains unclear.

The past decade’s research has increased comprehension of the adipose tissue’s role in cardiovascular disease. A large number of studies have confirmed that adipokines (cytokines specifically releasing from adipose tissue) exert protective or deleterious effects on cardiac functions [13, 14], especially current fascinating adipose-derived extracellular vesicles (EVs) and their cargoes in cardiac regulation (Table 1) [14–48]. Among them, the most concerned is adiponectin (APN), a well-known metabolic regulatory/cardioprotective adipokine [49]. Meanwhile, emerging evidence supports the existence of the “heart-adipose axis” in the human body [11]. It has been confirmed that ANP, which is a well-known cardiokine, regulates cardiac lipid metabolism through ANP receptors in cardiac tissue, including lipolysis, energy expenditure, and adipokine synthesis and secretion [50]. Under physiological conditions, baseline cardiokines contribute to maintaining metabolic homeostasis [51]. Under pathological conditions, damaged cardiac tissue-derived cardiokines contribute to metabolic disorders [52], such as heart failure [50], coronary heart disease [53], obesity and diabetes [54]. These data suggest bidirectional regulation between heart tissue and adipose tissue. Therefore, this review focuses on the current advances in cardiac-to-adipose communication to provide a novel therapeutic avenue for metabolic disorders caused by adipose dysfunction.

**Table 1:** Adipokines in cardiac regulation

Adipose location	Cellular type	Content/cargo	Healthy/pathological heart	Regulatory effects of cardiac function	Refs
Intrascapular BAT (iBAT)	Brown adipocyte	iNOS in sEV	Cardiac remodeling	Inducing the activation of cardiac fibroblasts (CFs) Protecting against cardiac remodeling	[16]
Intrascapular BAT (iBAT)	Brown adipocyte	miR-125b-5p, miR-128-3p, miR-30d-5p in sEV	MI/R	Ameliorating MI/R-related cardiac dysfunction Suppressing apoptosis by inhibition of MAPK pathway	[17]
Epididymal white adipose tissue (eWAT)	Adipocyte	miR-130b-3p in sEV	Diabetic MI/R	Increasing systolic/diastolic function Decreasing infarct size in cardiac tissue Promoting cardiomyocyte apoptosis	[18]
Pericardial adipose tissue	Adipocyte	Adipsin in sEV	MI	Alleviating MI-induced cardiac injury, including reduced myocardial fibrotic area and increased survival rate Mitigating iron over-loading and lipid oxidative stress	[19]
Epicardial fat (eFat)	Not mentioned	Cytokines, miRNAs in sEV	AF	Stimulating cardiac fibrosis Simulating angiogenesis by targeting endothelial cells Promoting the initiation and maintenance of reentrant arrhythmias	[20]
Not mentioned	Macrophage	miR-140-5p in sEV	Obesity-induced cardiac injury	Provoking obvious cardiac injury Inducing lipid peroxides and mitochondrial injury Promoting ferroptosis in cardiomyocytes	[21]
In vitro	3T3-L1 adipocyte	miR-802-5p in sEV	Neonatal rat ventricular myocytes	Inducing insulin resistance and mitigating the insulin-sensitizing effects of adiponectin Enhancing oxidative stress	[22]
In vitro	ADSC	SIRT1 in sEV	AMI	Increasing the survival rate Reducing infarct size and post-AMI left ventricular remodeling; Inducing vasculogenesis Decreasing AMI-induced myocardial inflammation; Promoting migration and tube formation of AMI-EPCs	[23]
In vitro	ADSC	miR-205 in sEV	AMI	Improving LVEF by alleviating MI-induced cardiac fibrosis Reducing cardiomyocyte apoptosis Increasing angiogenesis	[24]
In vitro	ADSC	miR-196a-5p, miR-425-5p in sEV	AMI	Preventing mitochondrial dysfunction and reactive oxygen species production Increasing angiogenesis Modulating macrophage polarization toward M2 Reducing myofibroblast activation and decreasing collagen expression	[25]
In vitro	ADSC	miR-320d in sEV	AF	Reducing apoptosis and increasing cell viability in cardiomyocytes Activating STAT3 signaling pathway	[26]
Adipose tissue	Adipocyte	APN	MI	Improving cardiac function	[15]
				Enhancing myocardial oxygen metabolism	[27]
				Decreasing LSG function and neural activity	[40]
				Suppressing ventricular arrhythmia	[41]
Adipose tissue	Adipocyte	omentin1	MI-induced HF	Ameliorating cardiac function, cardiac hypertrophy, infarct size and cardiac pathological features	[28]
				Increasing mitochondrial fusion and decreasing mitochondrial fission Promoting PINK1/Parkin-dependent mitophagy Enhancing SIRT3/FOXO3a signaling	[42]
Adipose tissue	Adipocyte	FABP4	Obese heart	Depressing shortening amplitude in cardiomyocytes Decreasing intracellular systolic peak Ca(2 +) in cardiomyocytes Reducing the excitation–contraction gain	[29, 43]
Adipose tissue	Adipocyte	Resistin	MI/R	Improving left ventricular ejection fraction Mitigating I/R-induced fibrosis; Reducing atrial natriuretic peptide/brain natriuretic peptide expression Inhibiting cardiomyocyte apoptosis Promoting ADSC proliferation	[30, 31, 44]
Adipose tissue	Adipocyte	Apelin	Pressure overload-heart	Preventing myocardial fibrosis and cardiac remodelling Inhibiting TGF-β1-mediated fibrotic response	[32, 45]
Adipose tissue	Adipocyte	CTRP3	Hypertension-induced cardiac hypertrophy	Restoring left ventricular cardiac contractile function Alleviating cardiac hypertrophy and fibrosis; Inhibiting expressions of hypertrophic and fibrotic signaling Modulating endoplasmic reticulum stress	[33, 34, 46]
Visceral adipose tissue	Adipocyte	TNF-α, visfatin, HMGB1	Obese cardiac cells (in *vitro*)	Provoking apoptosis Blocking differentiation	[35]
Visceral fat cell	Adipocyte	Vaspin	Sepsis-induced cardiac injury	Reducing mortality Alleviating cardiac injury and cardiac dysfunction Attenuating cardiac inflammation Reducing cardiomyocyte apoptosis	[36, 47]
Pericardial adipose tissue	Adipocyte	Leptin	HFD- induced obesity	Exacerbating myocardial remodeling and dysfunction Elevating oxidative stress and mitochondrial dysfunction in hearts Stimulating apoptosis of cardiomyoblasts	[37, 48]
Epididymal and pericardial adipose tissue	White adipocyte	SFRP5	MI/R	Restoring cardiac function Decreasing infarct size; Inhibiting cardiac myocyte apoptosis and inflammation	[38, 49]
Brown adipose tissue	Brown adipocyte	Neuregulin-4	Diabetic cardiomyopathy	Alleviating myocardial injury Upregulating autophagy via AMPK/mTOR pathway	[39]

*ADSCs* adipose-derived stem cells; *AF* Atrial fibrillation; *AMI* acute myocardial infarction; *APN* Adiponectin; *CTRP3* C1q-tumor necrosis factor-related protein-3; *BAT* brown adipose tissue; *EPCs* endothelial progenitor cells; *FABP4* fatty acid-binding protein; *HFD* high-fat diet; *iNOS* inducible nitric oxide synthase; *LSG*: left stellate ganglion; *LVEF* left ventricular ejection fraction; *MI* myocardial infarction; *MI-induced HF* myocardial ischemia-induced heart failure; *MI/R* myocardial ischemia/reperfusion; *MAPK* mitogen-associated protein kinase; *sEV* small extracellular vesicle; *SFRP5* secreted frizzled-related protein 5; *SIRT1* Sirtuin 1; *TGF-β1* transforming growth factor-β1

## The classification and endocrine manner of cardiokines

Hundreds of cardiokines that can be cardioprotective in an autocrine manner [55] or transferred to various peripheral organs [56], such as the spleen, kidney and skeletal muscle, have been identified, and dozens mediate interorgan interactions between the heart and adipose tissue. Cardiokines can be classified according to different standards. First, cardiokines can be produced and released by several cell types in cardiac tissue, including cardiomyocytes, fibroblasts, endothelial cells, cardiac progenitor cells, adipocytes and cardiac telocytes [57]. Among these, cardiomyocyte-derived cardiokines, which are known as cardiomyokines, are the most abundant, followed by fibroblasts and endothelial cell-derived cardiokines. Second, based on their different molecular structures, cardiokines can be divided into peptides, proteins, microRNAs, etc. Recently, extracellular vesicles (EVs) have been demonstrated to be involved in cardiac-adipose communication [58], and their cargos constitute cardiokines. Common cardiac peptides include natriuretic peptides, such as ANP and BNP [59]. Cardiac proteins have been studied frequently , including mediator complex subunit 13 (MED13), C1q/TNF-related proteins (CTRPs) [60], fibroblast growth factor 21 (FGF21) [61], FSTL1 and Mitsugumin 53 (MG53), which play vital roles in cardiac-adipose communication. Interestingly, microRNAs (miRNAs or miRs) [62], which are small noncoding RNAs that regulate gene expression, have been newly defined as cardiokines because of their enrichment in the heart. Common cardio-enriched miRNAs include miR-208a, miR-22-3p, miR-1956, miR-21-3p, and miR-409-3p [63]. Importantly, miRNA-mediated communication typically occurs in an EV-dependent manner. Common cardiokines with different molecular structures and cell sources are detailed in Table 2.

**Table 2:** Classification of cardiokines

Cellular type	Name	Structural type	Model	Genetic intervention	Effects on regulation of adipose function	Refs
Cardiomyocytes	ANP	Peptides	In vitro In vitro Human In vitro	No No No No	Improving glucose uptake and insulin sensitivity in AT Increasing energy expenditure and enhancing oxidative capacity in adipocytes Promoting the browning of WAT Regulating the balance between lipogenesis and lipolysis Influencing adipokines synthesis and secretion Regulating adipocyte differentiation and proliferation	[14, 49, 50, 57, 71,47. ]
	BNP	Peptides	Human Human	No No	Improving glucose uptake and insulin sensitivity in AT Increasing energy expenditure in adipocytes Promoting the browning of WAT Adipose tissue depots Regulating lipogenesis and lipolysis Influencing adipokines synthesis and secretion	[16, 34, 55, 56]
	MED13	Proteins	Mouse Rat Mouse	Yes No Yes	Gaining fat mass and body weight Improving systemic insulin sensitivity and glucose tolerance Increasing systematic energy expenditure Regulating WAT gene expression and promoting fatty acid oxidation	[42, 63, 76,46. ]
	CTRPs	Proteins	Mouse Mouse	Yes No	Improving glucose uptake and insulin sensitivity in AT Resisting weight gain and fat mass gain Increasing energy expenditure Promoting fatty acid oxidation Enhancing anti-contractile effect in AT	[35, 60, 89]
	FGF21	Proteins	Rat Mouse Mouse	No Yes Yes	Reducing body weight gain Regulating glucose metabolism and insulin sensitivity Increasing energy expenditure and fat utilization Increasing fatty acid oxidation, mitochondrial fat acid uptake and mitochondrial biogenesis Promoting the browning of WAT Influencing adipokines synthesis and secretion	[36, 65, [74]
	FSTL1	Proteins	In *vitro* Mouse Human	No Yes No	Regulating diet-induced systemic metabolism Influencing thermogenic ability in AT Promoting preadipocyte to adipocyte conversion	[45, 54, 70]
	MG53	Proteins	Mouse Mouse	No Yes	Regulating insulin resistance and glucose metabolism Promoting lipid utilization and FFA accumulation	[17, 61]
	MiR-208a	Micro RNA	Rat In *vitro*	No Yes	Controlling body weight gain and fat mass; Inducing mitochondrial β-oxidation	[63, 66]
	MiR-22-3p	Micro RNA	Human	No	Regulating cellular composition of the stromavascular VAT depot; Regulating adipose inflammation	[72]
	MiR-23–27-24	Micro RNA in sEV	Mouse	Yes	Regulating systemic metabolism; Suppressing adipocyte endocrine function; Attenuating adipocyte ER stress	[33]
	MiR-1956	Micro RNA in sEV	Mouse	Yes	Regulating cell proliferation of adipose-derived MSCs	[37]
Fibroblasts	MiR-21-3p	Micro RNA	Mouse	Yes	Regulating adipose browning; Down-regulating FGF21 expression	[44]
Endothelial cells	MiR-409-3p	Micro RNA	In *vitro*	Yes	Regulating glucose metabolism and insulin tolerance Increasing energy expenditure Decreasing expression of BAT markers Improving BAT angiogenesis	[77]

*ANP* atrial natriuretic peptide; *AT* adipose tissue; *BAT* brown adipose tissue; *BNP* brain natriuretic peptide; *CTRPs* C1q/TNF-related proteins; *ER* endoplasmic reticulum; *FFA* free fatty acid; *FGF21* fibroblast growth factor 21; *FSTL1* follistatin like 1; *MED13* mediator complex subunit 13; *MG53* mitsugumin 53; *MSC* mesenchymal stem cell; *VAT* visceral adipose tissue; *WAT* white adipose tissue

After synthesis and secretion in heart tissue, including cardiomyocytes and cardiac fibroblasts, cardiokines are thought to play roles in situ, in adjacent cells, or even over a long distance to maintain metabolic homeostasis and regulate metabolic disorders such as myocardial infarction (MI) and heart failure (HF) [64–66]. Here, we mainly discuss the endocrine effects of cardiac tissue and investigate the regulatory effects on adipose tissue; therefore, we focus on how cardiokines are transported to adipose tissue. Cardiokines are released into the blood and can be transported to terminal adipose tissue through the circulation, and an increase in circulating levels of cardiokines has been revealed [67]. Then, cardiokines are taken up by adipose tissue, especially adipocytes. Depending on the molecular types, cardiokines are accepted in different ways. Peptides and proteins such as NPs and FGF21 often bind with their specific receptors in adipose tissue, thus mediating subsequent cellular signaling [68, 69]. MicroRNAs are typically encapsulated in EVs and can be captured and endocytosed by adipocytes through membrane fusion [58]. Consequently, microRNAs are released into the cytoplasm and bind to the 3′UTR of specific mRNAs, resulting in the direct regulation of protein expression in adipose tissue [69].

## Cardiokines-mediated physiological and pathological communication

**Fig. 1:**
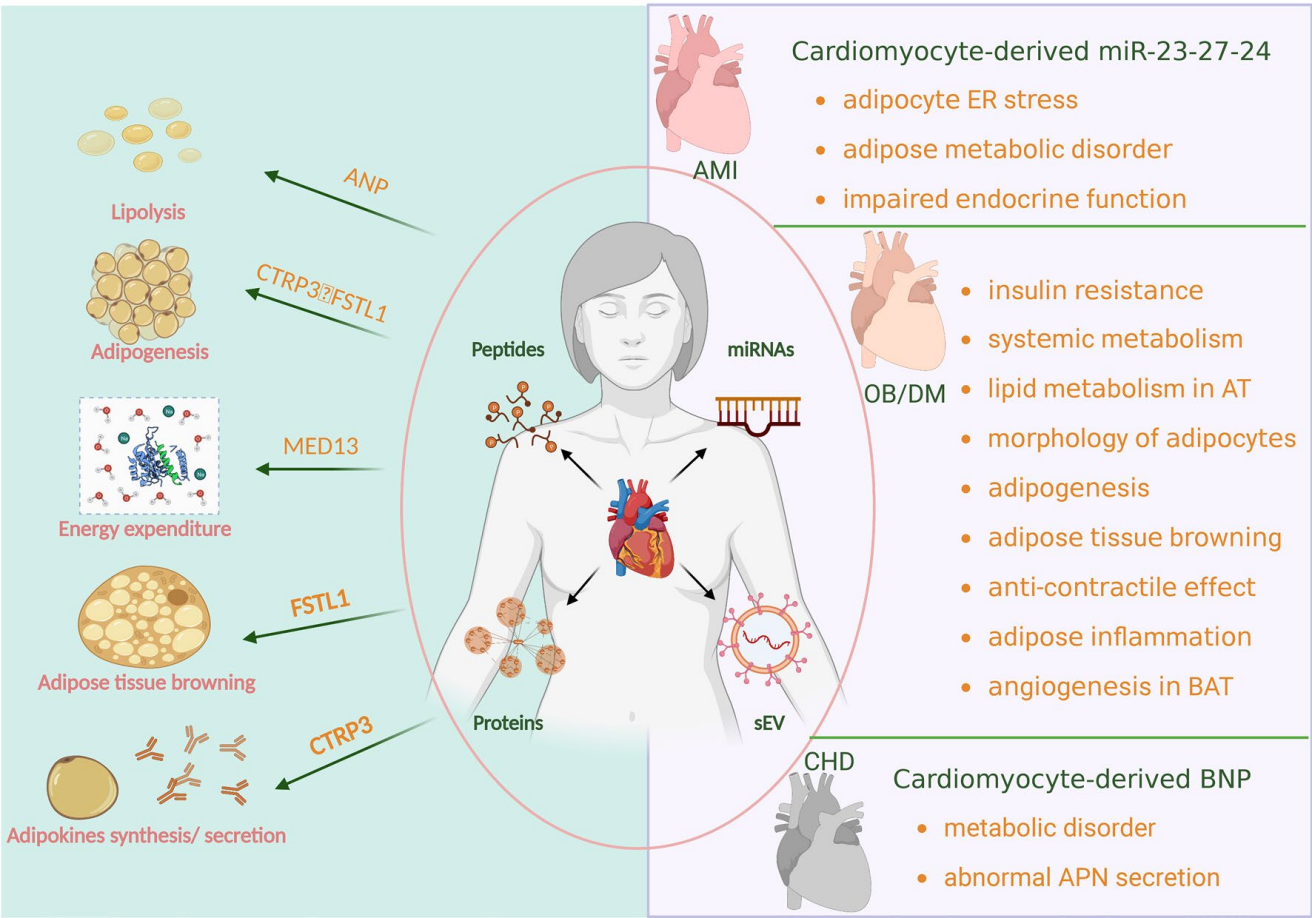
Cardiokines-mediated communication in physiological and pathological conditions. Cardiokines can be divided into peptides, proteins, miRNAs and sEVs. The physiological regulation of adipose tissue includes lipolysis, adipogenesis, energy expenditure, browning of adipose tissue, and the synthesis and secretion of adipokines. While in pathological situations, cardiokine-mediated adipose regulation differs in several diseases. The cardiomyocyte-derived miR-23-27-24 impairs adipose tissue's metabolism and endocrine function by exacerbating adipose ER stress in AMI. In CHD models, the cardiomyocyte-derived BNP leads to metabolic disorder and abnormal adiponectin secretion in adipose tissue. At the same time, adipokines in obesity and diabetes play a vital role in insulin resistance, systemic metabolism, lipid metabolism in AT, adipocyte cell size, adipogenesis, adipose tissue browning, the anti-contractile effect, adipose inflammation and angiogenesis in BAT

Cardiokines are transported to peripheral organs through the circulation and have various effects on physiological and pathological processes. These cardiokines include previously known adipokines and myokines, such as CTRP9, FGF21 and MG53. CTRPs and FGF21 were first discovered as adipocytokines with powerful regulatory effects on adipose metabolism. In recent years, CTRPs and FGF21 have been newly discovered in cardiac tissue, and there is evidence that these factors can be synthesized and secreted by cardiomyocytes (Fig. 1).

### Cardiokines maintain metabolic homeostasis

Under physiological conditions, cardiokine levels remain at baseline to maintain metabolic homeostasis, which is the physiological function of cardiokines, including regulation of lipolysis, adipogenesis, energy expenditure, and thermogenic activity in response to cold exposure and adipokine biosynthesis.

#### Enhancing adipose energy expenditure

Energy expenditure is essential for cellular energy homeostasis and prevents excessive nutrient accumulation that could lead to pathological conditions such as obesity and type 2 diabetes [70]. Cardiokines have been reported to modulate energy expenditure in adipose tissue which involves the proteins ANP, BNP and MED [71]. ANP improves energy expenditure, including oxygen utilization, by activating the AMPK pathway [72]. Moreover, cardiac-specific overexpression of MED13 enhances oxygen consumption and carbon dioxide production by upregulating multiple metabolic genes in the fatty acid β-oxidation pathway and the Krebs cycle, such as Slc27a2 and DLAT [67]. These results suggest that cardiac tissue physiologically regulates lipid metabolism and promotes systemic energy expenditure in adipose tissue through cardiokines.

#### Promoting lipolysis

Lipolysis is the hydrolysis of triacylglycerols (TGs), and adipose tissue is the most extensive storage site of TGs, suggesting that adipose tissue is the leading site for lipolysis [73]. Natriuretic peptides have been shown to regulate lipolysis in adipose tissue [51]. As a classical cardiomyokine, ANP modulates physiological lipid metabolism and oxygen utilization in adipose tissue, especially adipocytes [72, 74]. Beneficial effects on adipose metabolism, including increased lipolysis and mitochondrial oxidative capacity, are mediated by ANP in both human and mouse adipocytes, indicating the promotion of adipose metabolism in physiological situations.

#### Regulating adipogenesis

When nutrient and calorie intake exceeds energy expenditure, excess calories are stored in the adipose tissue through hyperplasia and hypertrophy. Hyperplasia, which is also known as adipogenesis, is a normal physiological process that promotes the conversion of perivascular preadipocytes into adipocytes [2]. Cardiac tissue has been observed to regulate adipogenesis in adipose tissue through these cardiac-derived proteins. Adipogenesis is stimulated by Ang II and suppressed by ANP at physiological concentrations [75]. MEDs are also involved in the regulation of adipogenesis. MED14 participates in PPARγ-dependent adipogenesis in mouse embryonic fibroblasts [76]. Moreover, MED23 and its transcription factor ELK1 regulate adipogenesis, and knockdown or antagonization of *Med23* or *Elk1* inhibits adipogenesis [77]. The newly identified cardiokine FSTL1-mediated adipogenesis was verified by its differential expression in preadipocytes and adipocytes [70]. Therefore, baseline adipogenesis could be physiologically regulated by cardiokines.

#### Strengthening adipose browning

Browning of adipose tissue refers to the switch of white adipocytes to brown fat cells, which are also known as beige cells [78], and these cells play a crucial role in regulating thermogenic activity and energy expenditure in adipose tissue. Cardiac tissue is reported to promote adipose browning through cardiokines and is associated with elevated levels of thermogenic gene expression and the consequent enhanced thermogenic activity. Cardiac FSTL1, especially glycosylated FSTL1, might play a major role in adipose browning in maintaining physiological thermogenic activity by activating the adrenergic receptor signaling pathway [79]. Although direct evidence of heart-specific deletion is lacking, cardiokines-mediated cardiac instruction is essential for physiological adipose tissue browning.

#### Enhancing adipokine biosynthesis

Adipokine biosynthesis is crucial for maintaining systemic metabolism. Emerging evidence has revealed the regulatory effects of cardiokines on adipokine synthesis and secretion. NPs increase the synthesis and secretion of adipokines in adipocytes [80, 81], indicating metabolic potency of cardiokines in adipose tissue. Both ANP and BNP enhanced the expression of adiponectin transcripts and it secretion in dose-dependent manners [82], and the high molecular-weight (HMW)-adiponectin levels in healthy subjects were elevated by ANP infusion [83]. All the regulations should be mediated by high-affinity transmembrane NP receptors, which expressed at high levels on adipocytes [81]. But the underling molecular mechanism need to be further elucidated.

### Cardiokines are involved in metabolic disorders

Contrary to physiological conditions, dysfunctional or injured cardiac tissue synthesizes and secretes abnormal levels of cardiokines, thus leading to disorders in adipose tissue, which are pathological effects of cardiokines under conditions such as obesity, diabetes, myocardial infarction, and coronary heart disease. These abnormal cardiokines include FGF21, miRNAs, EVs and the cargo released from injured cardiac tissue.

#### Cardiokines in obesity and diabetes

Obesity and type 2 diabetes are often caused by an overload of nutrients and calories, thus leading to subsequent multiorgan dysfunction. Several cardiokines derived from cardiac tissue have been reported to regulate systemic metabolism [84] and insulin resistance and affect adipocyte metabolic and endocrine functions.

1. Insulin resistance
  Insulin resistance and systemic metabolic disorder are the common manifestations of obesity and diabetes, which can be regulated by cardiokines. High-fat diet (HFD)-fed mice show significant insulin resistance and increased accumulation of lipid droplets in adipose tissue. Cardiomyokines such as ANP and CTRP9 attenuate HFD-induced glucose intolerance and insulin resistance, which are evaluated by IPGTT and ITT in mice, thus playing a protective role in HFD-induced obesity [85, 86]. Furthermore, the recently identified cardiokine MG53 might be deleterious to insulin resistance and metabolic syndrome and has a relatively high level in obesity and diabetes by promoting ubiquitin-dependent degradation of insulin receptor or insulin receptor substrate-1 [54]. The detrimental effect of MG53 on insulin resistance is confirmed by the worsening of insulin resistance after cardiac-specific overexpression of MG53 and improvements after treatment with the MG53-specific monoclonal antibody [87]. These results suggest the regulatory effects of cardiokines on insulin resistance and systemic metabolic syndrome and provide potential therapeutic directions in obesity and diabetes.
2. Adipocyte hypertrophy
  Adipocyte hypertrophy is more significant in obesity and diabetes, and there is increased accumulation of lipid droplets, suggesting impaired lipid metabolism in adipocytes. MED13 participates in pathological adipocyte hypertrophy, and lower MED13 level has been observed in obesity and diabetes. In addition, cardiac-specific ablation of MED13 increases susceptibility to obesity [88], while cardiac overexpression of MED13 leads to a lean phenotype [67], which shows the crucial role of MED13 in obesity. MiR-208a is a heart-specific miRNA that acts as a cardiokine [89], in addition to cardiac miR-378 and miR-378* [90]. Its expression increases with adipocyte hypertrophy and obesity, and it plays a role in adipose regulation by inhibiting MED13 expression [91]. However, a decrease in miR-208a expression increases body weight gain, WAT mass and adipocyte size in adipocytes [92]. These conflicting results may be attributed to the differences in target molecules of miR-208a, which are MED13 dependent or independent [89]. Therefore, targeting MED13 and its upstream regulators might be beneficial for inhibiting pathological adipocyte hypertrophy in obesity and diabetes.
3. Impaired adipogenesis
  Significantly impaired adipogenesis has been observed in obesity and diabetes, and CTRP6 and FSTL1 are involved in pathological adipogenesis. Elevated CTRP6 expression is observed in adipose tissue during obesity, which is accompanied by inhibited adipogenesis [93]. CTRP6-mediated adipogenesis involves in the inhibition of adipocyte differentiation after CTRP6 overexpression and the restoration after CTRP6 ablation in a PPAR-γ-dependent manner, indicating the possible protective effects of CTRP6 on obesity and diabetes, although CTRP6 depletion is not cardiac specific. In contrast, cardiac FSTL1 has the opposite effect as CTRP6, and an increase in FSTL1 promotes preadipocyte-to-adipocyte conversion [94] and enhances adipogenesis in obesity and diabetes [95]. A decrease in FSTL1 expression is associated with a reduction in adipogenesis in severe obesity, which is accompanied more severe senescence in preadipocytes and increased apoptosis in adipocytes [96], which conflicts with previous results. A possible explanation may be that FSTL1 is exhausted by the significant senescence of the accumulated preadipocytes in extreme obesity, which leads to exacerbated apoptosis in adipocytes.
4. Limited adipose browning
  Recent studies have demonstrated that impaired adipose tissue browning in obesity and type 2 diabetes while promoting adipose tissue browning can attenuate pathological changes [78]. UCP1 is highly specific in brown adipose tissue and indicates a change in adipose browning. Cardiomyocyte-derived ANP and fibroblast-derived miR-21-3p are involved in the rebrowning of adipose tissue in HFD-fed mice. ANP attenuates HFD-induced BAT whitening by promoting UCP1 expression through activation of the p38 MAPK or GPR120 pathway [85, 97], while miR-21-3p, which directly targets FGFR1 in adipose tissue, inhibits adipose rebrowning by repressing UCP1 expression in HFD [69]. Consequently, further ANP administration and specific antagonism of miR-21-3p or blockade of miR-21-3p-FGFR1 binding might be effective approaches to promote adipose browning and ameliorate HFD-induced pathological processes.
5. Adipose inflammation
  Chronic systemic inflammation is an essential feature of obesity and type 2 diabetes and can manifest as adipose inflammation [19], which is modulated by cardiac tissue in an endocrine manner. Cardiokines such as FSTL1 and miR-22-3p exhibit high pathological levels in obesity and diabetes, leading to an abundant proinflammatory profile including IL-6, TNF-α and MCP-1 [95, 98]. In contrast, in addition to its adipogenic properties, CTRP6 is a proinflammatory cardiomyokine in obese adipose tissue, and an increase in CTRP6 induces the expression of inflammatory genes such as *Tnf-α*, *Ccl2* and *Il6*, which is further evidenced by gain- and loss-of-function experiments [93]. Therefore, inhibiting proinflammatory cardiokines or promoting anti-inflammatory cardiokines might be beneficial to adipose homeostasis and would be a novel therapeutic strategy in obesity and diabetes.

#### Cardiokines derived from ischemic hearts induce adipose dysfunction

Clinical studies have shown that patients with heart failure have local and systemic metabolic disorders, which are characterized by progressive wasting, adipocyte size alternation, and adipokine biosynthesis disorders. Ventricular assist device implantation to restore cardiac function significantly corrects metabolic disorders and adipose dysfunction, indicating cardiac instruction to adipose tissue under pathological conditions [52]. The most striking phenomenon is the change of adiponectin (APN) biosynthesis after ischemic heart disease. Lower plasma APN levels were observed in patients with coronary artery disease and acute myocardial infarction, correlated with worse cardiac functional recovery after MI with reperfusion [99–101], indicating adipocyte endocrine dysfunction and impaired APN secretion occurred after acute cardiac injury. However, the hypoadiponectinemia usually accompany with heart failure, and hyperadiponectinemia is associated with poor cardiac function and increased mortality in these patient populations [102, 103]. The mechanism involved in the regulation of adiponectin biosynthesis after acute and chronic ischemic heart disease has not been clearly explained. Recently, our group demonstrated myocardial miR-23–27-24 contributed to the reduced circulating APN levels after MI/R [58], which provided the first evidence that cardiac factors directly lead to adipocyte endocrine dysfunction after ischemic heart injury, and supported the importance of cardiac-to-adipose communication.

In addition, increased serum levels of FGF21 have been observed in patients with acute myocardial infarction (AMI), along with higher serum fatty acid binding protein-4 (FABP4) and saturated fatty acid levels [104]. In coronary heart disease patients with heart failure, higher serum BNP levels are commonly associated with higher circulating adiponectin levels [53]. Additionally, FGF21 expression is elevated in cardiomyocytes after cardiac endoplasmic reticulum (ER) stress [105], which is accompanied by defective lipolysis and disturbed energy homeostasis [106]. These results suggest that adipose dysfunction are closely related with ischemic heart injury, while cardiokines are responsible for heart-to-adipose communication.

## Regulatory signaling pathways of cardiokines

**Fig. 2:**
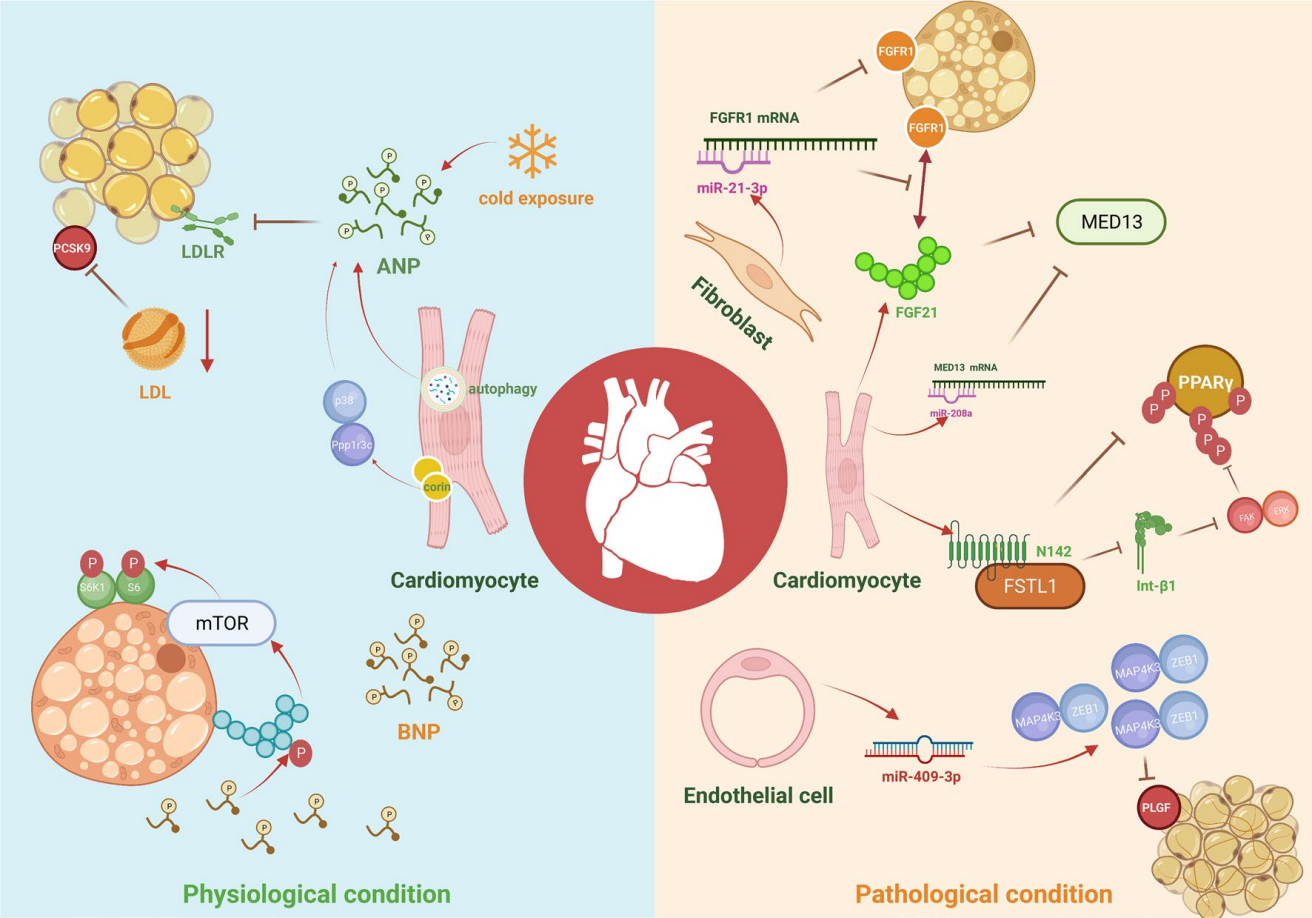
Underlying upstream and downstream mechanisms of cardiokines that mediate the heart-to-adipose tissue communication. Under physiological conditions, cardiomyocytes are the primary source of cardiokines, including the cardiomyocyte-derived ANP and BNP. Autophagy and cardiac corin regulators ANP synthesis and secretion in cardiomyocytes. When exposed to cold temperatures, ANP down-regulates LDL metabolism by decreasing adipocyte LDLR through the PCSK1 pathway. Meanwhile, cardiomyocyte-derived BNP increases mTORC1 expression and promotes the phosphorylation of S6K1 and S6 through the PKG/p-PKG signaling pathway. While in pathological conditions, cardiokines are derived from cardiomyocytes, cardiac fibroblasts and endothelial cells. EC-mediated miR-409-3p inhibits PLGF expression and angiogenesis in BAT through ZEB1 and MAP4K3 activation. Fibroblast-mediated miR-21-3p suppresses the FGFR1 in adipocytes and impairs the combination of FGF21-FGFR1 through direct binding with FGFR1 mRNA. In addition, the cardiomyocyte-derived FSTL1 inhibits the phosphorylation of PPARγ through the integrin β1-FAK/ERK signaling by the N-glycosylation of FSTL1 in the N142 site. Cardiomyocyte-derived miR-208a decreases MED13 expression by inhibiting MED13 mRNA translation through miR-208a-MED13 mRNA combination

Presently, the signaling pathway of cardiokines-mediated heart-adipose tissue communication is not fully understood. Herein, we describe recent research investigating the underlying mechanisms of cardiokines such as ANP, BNP, FGF21, MED13, FSTL1 and miRNAs (Fig. 2), including the cell proliferation- and differentiation-associated, autophagy-associated, inflammation-associated, and exocytosis-associated signaling pathways.

### Cell proliferation-associated signaling pathways

Adipocyte proliferation, which is also named adipogenesis, is predominant in the pathological process of obesity and obesity-related diseases, and MED13 levels are negatively correlated with adipocyte proliferation in HFD-induced obesity [91]. Kruppel-like factor 5 (KLF5) can upregulate MED13, as it can directly bind to the *Med13* promoter and promote MED13 expression. Furthermore, miRNA-208a downregulates MED13 by binding with the MED13 mRNA 3′UTR [89, 107] in obesity and diabetes. However, little is known about the relationship between KLF5 and miRNA-208a in regulating MED13-mediated adipocyte proliferation and whether they interact with each other or exert their effects independently. In addition, the EC-derived cardiokine miR-409-3p plays an essential role in EC-BAT crosstalk and inhibits angiogenesis in BAT through the ZEB1/MAP4K3 pathway [108].

### Cell differentiation-associated signaling pathways

Adipocyte differentiation, which contributes to adipose browning, regulates the metabolic and endocrine functions of adipose tissue, and PPARγ is a prominent signaling pathway [109]. Recently, there have been several upstream regulators of PPARγ, including FGF21 and FSTL1. First, elevated levels of FGF21 in ischemic heart injury, including myocardial infarction, inhibit the phosphorylation of PPARγ, which is the active form of PPARγ, thus suppressing adipose browning in WAT [91]. Additionally, the regulatory effect of FGF21 is partly dependent on cardiomyocyte-derived KLF5, as evidenced by cardiomyocyte-specific KLF5-knockout mice. Moreover, cardiac fibroblast-derived miR-21-3p can negatively regulate the expression of FGFR1, the molecule that binds to FGF21, by binding to the 3′UTR of *Fgfr1* mRNA [69]. This FGF21-FGFR1 mismatch eventually leads to adipose dysfunction. Second, apart from promoting adipogenesis in adipose tissue, N142-glycosylated FSTL1 is essential for PPARγ activation and subsequently leads to adipocyte differentiation into brown adipose tissue [110]. In addition, the biological effects of glycosylated FSTL1 might rely on the integrin β1-FAK/ERK pathway, but the present evidence is limited, and further research is needed.

### Autophagy-associated signaling pathway

Autophagy plays a central role in cardiokine-mediated myocardial-adipose crosstalk in physiological and pathological situations. On the one hand, cardiac autophagy, especially cardiomyocyte-specific autophagy, regulates the physiological synthesis and secretion of cardiokines such as ANP and BNP [111]. On the other hand, cardiokines can activate the mTOR signaling pathway via the phosphorylation of S6K1 and S6, which are the downstream targets of mTOR, which is essential for autophagy inhibition [112]. Thus, baseline autophagy maintains baseline levels of cardiokines under physiological homeostasis. In general, MAPK acts as an important upstream regulator of cardiac autophagy and subsequent cardiokine-mediated thermogenesis through activation of the p38-MAPK/Ppp1r3c pathway [85]. Moreover, there are several downstream regulators of autophagy, including PCSK9, the alpha2 subunit of AMPK, and phosphorylated PKG, that regulate lipoprotein metabolism, lipolysis and adipose browning in adipose tissue [72, 112, 113].

### Inflammation-associated signaling pathways

Excessive adipose inflammation is a vital feature of obesity and type 2 diabetes, and the SIRT1 pathway is essential in adipose inflammation. Moreover, clinical and experimental studies have demonstrated that overexpression of the cardiokine miR-22-3p might upregulate SIRT1 inhibition in a severely inflamed profile in diabetes [98]. Unfortunately, gain- and loss-of-function models are lacking, and further research is needed.

### Exocytosis-associated signaling pathways

Aberrant exocytosis promotes interorgan crosstalk via vesicle content release through the fusion of the vesicle membrane with the plasma membrane, which depends on cytosolic Ca^2+^ concentrations. The high glucose-induced increase in the expression of the SNARE-binding protein synaptotagmin 7 (Syt7), which is an important Ca^2+^ sensor, is responsible for the higher levels of cytosolic Ca^2+^ and the increased release of glucose-triggered MG53 via exocytosis [54].

## Extracellular vesicle-mediated heart-to-adipose communication

Extracellular vesicles (EVs) are systemic messengers that deliver signaling molecules that mediate intracellular and intraorgan communication. Exosomes and microvesicles, which have different biogenesis pathways and are collectively known as small EVs (sEVs), are two of the most important EV types. sEV biogenesis is greatly enhanced after heart injury [114]. Our group demonstrated that small extracellular vesicles (sEVs) from injured mouse cardiomyocytes after myocardial ischemia/reperfusion (MI/R), which exert cardiokine-like effects, induced adipocyte ER stress and adipokine biosynthesis disorders in adipose droplets [58]. Inhibition of cardiac sEV biogenesis by GW4869 administration alleviates adipocyte ER stress and restores adipose metabolic and endocrine functions. Further analysis indicates that miR-23-27-24 is an effective molecule in cardiomyocyte-derived sEVs after MI/R that induces adipose dysfunction by suppressing EDEM3 expression. Moreover, cardiac sEVs carrying miR-1956 from the ischemic heart stimulate adipose-derived mesenchymal stem cell-mediated proangiogenic paracrine signaling by suppressing Notch-1. These results suggest that EVs, which are essential components of the myocardial secretome, affect adipose function by delivering messengers (cardiokines) from the heart to adipose tissue. In addition, with the progress in sEV isolation and research technology, more powerful evidence will be obtained on the regulation of cardiac sEV generation, cargo sorting and packaging, and vesicle orientation intervention.

## Cardiokine-mediated clinical therapy

Although numerous animal experiments have investigated the regulatory effects of various kinds of cardiokines on metabolic and endocrine functions in adipose tissue, limited clinical data are available for cardiokine-mediated therapy in patients. Natriuretic peptides, including ANP and BNP, are the only cardiokines under clinical investigation. NPs enhance lipid metabolism and energy expenditure both in healthy individuals [115] and in heart failure (HF) patients [116], indicating their advantages in maintaining metabolic homeostasis and improving pathological metabolic disorders. Moreover, endocrine function is associated with human ANP administration in patients with congestive heart failure (CHF), which is characterized by a significantly increase in adiponectin in plasma after ANP management in CHF patients [82]. There are two main reasons why NPs are the only cardiokines used in the clinic to date. First, NPs are well known, as many animal models have been examined; thus, they could be applied in the clinic. Second, natriuretic peptide receptors (NPRs) are found in human adipose tissue [117], and inhibiting natriuretic peptide clearance receptors seems beneficial to diabetic patients [118], which demonstrates the pivotal role of NPRs in adipose regulation. However, the present clinical studies are limited, and more research regarding other cardiokines is needed.

## Conclusion and prospects

In conclusion, cardiokines widely participate in metabolic and endocrine regulation of adipose tissue, including weight gain, systemic metabolism (glucose level, insulin sensitivity, lipolysis), fat mass, adipocyte size, adipogenesis, preadipocyte to adipocyte conversion, adipose browning, and the synthesis and secretion of adipokines. Unfortunately, these studies have focused almost exclusively on the roles of cardiokines on classical white and brown adipose depots, ignoring the adipose tissue with special locations, such as epicardial adipose tissue, pericardial adipose tissue and perivascular adipose tissue. Due to its complex structure and numerous component cell types, their biological roles vary greatly. Therefore, the future studies should fucus on revealing the effect of cardiokines on these special adipose depots. Moreover, we supported that cardiokines played essential roles in adipose metabolism in populations with obesity, type 2 diabetes mellitus (T2DM) and other metabolic-associated diseases, and feedback exacerbated ischemic heart injury through adipokine-mediated instruction [121], forming a vicious cycle. Supplementation or inhibition of specific cardiokines may change the metabolic status in individuals with obesity and diabetes, thus providing a promising treatment strategy. Moreover, in the treatment of heart diseases, blocking cardiokine-mediated adipose dysfunction has a positive effect on reducing cardiac injury and improving prognosis. However, these conclusions are based only on studies in animal models. More research, especially clinical research, is needed in the future.

## Data Availability

Not applicable.

## Abbreviations

ADSCs: Adipose-derived stem cells
AF: Atrial fibrillation
AMI: Acute myocardial infarction
ANF: Atrial natriuretic factor
Ang II: Angiotensin II
ANP: Atrial natriuretic peptide
APN: Adiponectin
AT: Adipose tissue
BAT: Brown adipose tissue
BNP: Brain natriuretic peptide
CTRPs: C1q/TNF-related proteins
EPCs: Endothelial progenitor cells
ER: Endoplasmic reticulum
FABP4: Fatty acid-binding protein
FFA: Free fatty acid
FGF21: Fibroblast growth factor 21
FSTL1: Follistatin like 1
HF: Heart failure
HFD: High-fat diet
iNOS: Inducible nitric oxide synthase
KLF5: Kruppel-like factor 5
LSG: Left stellate ganglion
LVEF: Left ventricular ejection fraction
MAPK: Mitogen-associated protein kinase
MED13: Mediator complex subunit 13
MG53: Mitsugumin 53
MI: Myocardial infarction
MI-induced HF: Myocardial ischemia-induced heart failure
MI/R: Myocardial ischemia/reperfusion
MSC: Mesenchymal stem cell
sEV: Small extracellular vesicle
SFRP5: Secreted frizzled-related protein 5
SIRT1: Sirtuin 1
TGs: Triacylglycerols
TGF-β1: Transforming growth factor-β1
TNF-α: Tumor necrosis factor-α
VAT: Visceral adipose tissue
WAT: White adipose tissue

## References

[1] Whitehead A, Krause FN, Moran A, MacCannell ADV, Scragg JL, McNally BD (2021). Brown and beige adipose tissue regulate systemic metabolism through a metabolite interorgan signaling axis. Nat Commun.

[2] Ghaben AL, Scherer PE (2019). Adipogenesis and metabolic health. Nat Rev Mol Cell Biol.

[3] Suchacki KJ, Cawthorn WP, Rosen CJ (2016). Bone marrow adipose tissue: formation, function and regulation. Curr Opin Pharmacol.

[4] Frühbeck G, Méndez-Giménez L, Fernández-Formoso JA, Fernández S, Rodríguez A (2014). Regulation of adipocyte lipolysis. Nutr Res Rev.

[5] Song T, Yang Y, Zhou Y, Wei H, Peng J (2017). GPR120: a critical role in adipogenesis, inflammation, and energy metabolism in adipose tissue. Cell Mol Life Sci.

[6] Lee MJ (2018). Transforming growth factor beta superfamily regulation of adipose tissue biology in obesity. Biochim Biophys Acta Mol Basis Dis.

[7] Infante M, Armani A, Mammi C, Fabbri A, Caprio M (2017). Impact of adrenal steroids on regulation of adipose tissue. Compr Physiol.

[8] Kim JH, Cho HT, Kim YJ (2014). The role of estrogen in adipose tissue metabolism: insights into glucose homeostasis regulation. Endocr J.

[9] Maurizi G, Babini L, Della GL (2018). Potential role of microRNAs in the regulation of adipocytes liposecretion and adipose tissue physiology. J Cell Physiol.

[10] Wu YS, Zhu B, Luo AL, Yang L, Yang C (2018). The role of cardiokines in heart diseases: beneficial or detrimental?. Biomed Res Int.

[11] Nakamura M, Sadoshima J (2014). Heart over mind: metabolic control of white adipose tissue and liver. EMBO Mol Med.

[12] Kivelä R, Hemanthakumar KA, Vaparanta K, Robciuc M, Izumiya Y, Kidoya H (2019). Endothelial cells regulate physiological cardiomyocyte growth via VEGFR2-mediated paracrine signaling. Circulation.

[13] Smith CC, Yellon DM (2011). Adipocytokines, cardiovascular pathophysiology and myocardial protection. Pharmacol Ther.

[14] Zhou Z, Liu C, Xu S, Wang J, Guo F, Duan S (2022). Metabolism regulator adiponectin prevents cardiac remodeling and ventricular arrhythmias via sympathetic modulation in a myocardial infarction model. Basic Res Cardiol.

[15] Lin JR, Ding LL, Xu L, Huang J, Zhang ZB, Chen XH (2022). Brown adipocyte ADRB3 mediates cardioprotection via suppressing exosomal iNOS. Circ Res.

[16] Zhao H, Chen X, Hu G, Li C, Guo L, Zhang L (2022). Small extracellular vesicles from brown adipose tissue mediate exercise cardioprotection. Circ Res.

[17] Man W, Song X, Xiong Z, Gu J, Lin J, Gu X (2022). Exosomes derived from pericardial adipose tissues attenuate cardiac remodeling following myocardial infarction by adipsin-regulated iron homeostasis. Front Cardiovasc Med.

[18] Shaihov-Teper O, Ram E, Ballan N, Brzezinski RY, Naftali-Shani N, Masoud R (2021). Extracellular vesicles from epicardial fat facilitate atrial fibrillation. Circulation.

[19] Zhao X, Si L, Bian J, Pan C, Guo W, Qin P (2022). Adipose tissue macrophage-derived exosomes induce ferroptosis via glutathione synthesis inhibition by targeting SLC7A11 in obesity-induced cardiac injury. Free Radic Biol Med.

[20] Wen Z, Li J, Fu Y, Zheng Y, Ma M, Wang C (2020). Hypertrophic adipocyte-derived exosomal mir-802-5p contributes to insulin resistance in cardiac myocytes through targeting HSP60. Obesity.

[21] Huang H, Xu Z, Qi Y, Zhang W, Zhang C, Jiang M (2020). Exosomes from SIRT1-overexpressing adscs restore cardiac function by improving angiogenic function of EPCs. Mol Ther Nucleic Acids.

[22] Wang T, Li T, Niu X, Hu L, Cheng J, Guo D (2023). ADSC-derived exosomes attenuate myocardial infarction injury by promoting miR-205-mediated cardiac angiogenesis. Biol Direct.

[23] de Almeida Oliveira NC, Neri EA, Silva CM, Valadão IC, Fonseca-Alaniz MH, Zogbi C (2022). Multicellular regulation of miR-196a-5p and miR-425-5 from adipose stem cell-derived exosomes and cardiac repair. Clin Sci.

[24] Liu L, Zhang H, Mao H, Li X, Hu Y (2019). Exosomal miR-320d derived from adipose tissue-derived MSCs inhibits apoptosis in cardiomyocytes with atrial fibrillation (AF). Artif Cells Nanomed Biotechnol.

[25] Gan L, Xie D, Liu J, Bond Lau W, Christopher TA, Lopez B (2020). Small extracellular microvesicles mediated pathological communications between dysfunctional adipocytes and cardiomyocytes as a novel mechanism exacerbating ischemia/reperfusion injury in diabetic mice. Circulation.

[26] Grossini E, Prodam F, Walker GE, Sigaudo L, Farruggio S, Bellofatto K (2014). Effect of monomeric adiponectin on cardiac function and perfusion in anesthetized pig. J Endocrinol.

[27] Hu J, Liu T, Fu F, Cui Z, Lai Q, Zhang Y (2022). Omentin1 ameliorates myocardial ischemia-induced heart failure via SIRT3/FOXO3a-dependent mitochondrial dynamical homeostasis and mitophagy. J Transl Med.

[28] Lamounier-Zepter V, Look C, Alvarez J, Christ T, Ravens U, Schunck WH (2009). Adipocyte fatty acid-binding protein suppresses cardiomyocyte contraction: a new link between obesity and heart disease. Circ Res.

[29] He Y, Guo Y, Xia Y, Guo Y, Wang R, Zhang F (2019). Resistin promotes cardiac homing of mesenchymal stem cells and functional recovery after myocardial ischemia-reperfusion via the ERK1/2-MMP-9 pathway. Am J Physiol Heart Circ Physiol.

[30] Zhao B, Bouchareb R, Lebeche D (2022). Resistin deletion protects against heart failure injury by targeting DNA damage response. Cardiovasc Res.

[31] Pchejetski D, Foussal C, Alfarano C, Lairez O, Calise D, Guilbeau-Frugier C (2012). Apelin prevents cardiac fibroblast activation and collagen production through inhibition of sphingosine kinase 1. Eur Heart J.

[32] Yi W, Sun Y, Yuan Y, Lau WB, Zheng Q, Wang X (2012). C1q/tumor necrosis factor-related protein-3, a newly identified adipokine, is a novel antiapoptotic, proangiogenic, and cardioprotective molecule in the ischemic mouse heart. Circulation.

[33] Zhang B, Zhang P, Tan Y, Feng P, Zhang Z, Liang H (2019). C1q-TNF-related protein-3 attenuates pressure overload-induced cardiac hypertrophy by suppressing the p38/CREB pathway and p38-induced ER stress. Cell Death Dis.

[34] Pérez LM, de Lucas B, Bernal A, Gálvez BG (2020). Adipokines disrupt cardiac differentiation and cardiomyocyte survival. Int J Obes.

[35] Yin N, Pan F, Qiu L, Yang Z, Xiong R, Shi L (2022). Vaspin alleviates sepsis-induced cardiac injury and cardiac inflammation by inhibiting kallikrein 7 in mice. Mediators Inflamm.

[36] Wang P, Luo C, Zhu D, Song Y, Cao L, Luan H (2021). Pericardial adipose tissue-derived leptin promotes myocardial apoptosis in high-fat diet-induced obese rats through janus kinase 2/reactive oxygen species/Na + /K + − ATPase signaling pathway. J Am Heart Assoc.

[37] Nakamura K, Sano S, Fuster JJ, Kikuchi R, Shimizu I, Ohshima K (2016). Secreted frizzled-related protein 5 diminishes cardiac inflammation and protects the heart from ischemia/reperfusion injury. J Biol Chem.

[38] Wang H, Wang L, Hu F, Wang P, Xie Y, Li F (2022). Neuregulin-4 attenuates diabetic cardiomyopathy by regulating autophagy via the AMPK/mTOR signalling pathway. Cardiovasc Diabetol.

[39] Chen WJ, Rijzewijk LJ, van der Meer RW, Heymans MW, van Duinkerken E, Lubberink M (2011). Association of plasma osteoprotegerin and adiponectin with arterial function, cardiac function and metabolism in asymptomatic type 2 diabetic men. Cardiovasc Diabetol.

[40] Jenke A, Wilk S, Poller W, Eriksson U, Valaperti A, Rauch BH (2013). Adiponectin protects against toll-like receptor 4-mediated cardiac inflammation and injury. Cardiovasc Res.

[41] Jin Z, Xia F, Dong J, Lin T, Cai Y, Chen J (2021). Omentin-1 attenuates glucocorticoid-induced cardiac injury by phosphorylating GSK3β. J Mol Endocrinol.

[42] von Jeinsen B, Ritzen L, Vietheer J, Unbehaun C, Weferling M, Liebetrau C (2020). The adipokine fatty-acid binding protein 4 and cardiac remodeling. Cardiovasc Diabetol.

[43] McManus DD, Lyass A, Ingelsson E, Massaro JM, Meigs JB, Aragam J (2012). Relations of circulating resistin and adiponectin and cardiac structure and function: the framingham offspring study. Obesity.

[44] El Mathari B, Briand P, Corbier A, Poirier B, Briand V, Raffenne-Devillers A (2021). Apelin improves cardiac function mainly through peripheral vasodilation in a mouse model of dilated cardiomyopathy. Peptides.

[45] Ma ZG, Yuan YP, Xu SC, Wei WY, Xu CR, Zhang X (2017). CTRP3 attenuates cardiac dysfunction, inflammation, oxidative stress and cell death in diabetic cardiomyopathy in rats. Diabetologia.

[46] Zhang D, Zhu H, Zhan E, Wang F, Liu Y, Xu W (2021). Vaspin mediates the intraorgan crosstalk between heart and adipose tissue in lipoatrophic mice. Front Cell Dev Biol.

[47] Omoto ACM, do Carmo JM, Nelson B, Aitken N, Dai X, Moak S (2022). Central nervous system actions of leptin improve cardiac function after ischemia-reperfusion: roles of sympathetic innervation and sex differences. J Am Heart Assoc..

[48] Tong S, Du Y, Ji Q, Dong R, Cao J, Wang Z (2020). Expression of Sfrp5/Wnt5a in human epicardial adipose tissue and their relationship with coronary artery disease. Life Sci.

[49] Villarreal-Molina MT, Antuna-Puente B (2012). Adiponectin: anti-inflammatory and cardioprotective effects. Biochimie.

[50] Jahng JW, Song E, Sweeney G (2016). Crosstalk between the heart and peripheral organs in heart failure. Exp Mol Med.

[51] Coué M, Moro C (2016). Natriuretic peptide control of energy balance and glucose homeostasis. Biochimie.

[52] Khan RS, Kato TS, Chokshi A, Chew M, Yu S, Wu C (2012). Adipose tissue inflammation and adiponectin resistance in patients with advanced heart failure: correction after ventricular assist device implantation. Circ Heart Fail.

[53] Antonopoulos AS, Margaritis M, Coutinho P, Digby J, Patel R, Psarros C (2014). Reciprocal effects of systemic inflammation and brain natriuretic peptide on adiponectin biosynthesis in adipose tissue of patients with ischemic heart disease. Arterioscler Thromb Vasc Biol.

[54] Wu HK, Zhang Y, Cao CM, Hu X, Fang M, Yao Y (2019). Glucose-sensitive myokine/cardiokine mg53 regulates systemic insulin response and metabolic homeostasis. Circulation.

[55] Doroudgar S, Glembotski CC (2011). The cardiokine story unfolds: ischemic stress-induced protein secretion in the heart. Trends Mol Med.

[56] Planavila A, Fernández-Solà J, Villarroya F (2017). Cardiokines as modulators of stress-induced cardiac disorders. Adv Protein Chem Struct Biol.

[57] Xu MY, Ye ZS, Song XT, Huang RC (2019). Differences in the cargos and functions of exosomes derived from six cardiac cell types: a systematic review. Stem Cell Res Ther.

[58] Gan L, Liu D, Xie D, Bond Lau W, Liu J, Christopher TA (2022). Ischemic heart-derived small extracellular vesicles impair adipocyte function. Circ Res.

[59] Katsi V, Marketou M, Antonopoulos AS, Vrachatis D, Parthenakis F, Tousoulis D (2019). B-type natriuretic peptide levels and benign adiposity in obese heart failure patients. Heart Fail Rev.

[60] Yang Y, Li Y, Ma Z, Jiang S, Fan C, Hu W (2016). A brief glimpse at CTRP3 and CTRP9 in lipid metabolism and cardiovascular protection. Prog Lipid Res.

[61] Suassuna PGA, Cherem PM, de Castro BB, Maquigussa E, Cenedeze MA, Lovisi JCM (2020). αKlotho attenuates cardiac hypertrophy and increases myocardial fibroblast growth factor 21 expression in uremic rats. Exp Biol Med (Maywood).

[62] Gao L, Mei S, Zhang S, Qin Q, Li H, Liao Y (2020). Cardio-renal exosomes in myocardial infarction serum regulate proangiogenic paracrine signaling in adipose mesenchymal stem cells. Theranostics.

[63] Vienberg S, Geiger J, Madsen S, Dalgaard LT (2017). MicroRNAs in metabolism. Acta Physiol.

[64] Yan W, Guo Y, Tao L, Lau WB, Gan L, Yan Z (2017). C1q/tumor necrosis factor-related protein-9 regulates the fate of implanted mesenchymal stem cells and mobilizes their protective effects against ischemic heart injury via multiple novel signaling pathways. Circulation.

[65] Masurkar N, Bouvet M, Logeart D, Jouve C, Dramé F, Claude O (2023). Novel cardiokine GDF3 predicts adverse fibrotic remodeling after myocardial infarction. Circulation.

[66] Askevold ET, Aukrust P, Nymo SH, Lunde IG, Kaasbøll OJ, Aakhus S (2014). The cardiokine secreted Frizzled-related protein 3, a modulator of Wnt signalling, in clinical and experimental heart failure. J Intern Med.

[67] Baskin KK, Grueter CE, Kusminski CM, Holland WL, Bookout AL, Satapati S (2014). MED13-dependent signaling from the heart confers leanness by enhancing metabolism in adipose tissue and liver. EMBO Mol Med.

[68] Bordicchia M, Liu D, Amri EZ, Ailhaud G, Dessì-Fulgheri P, Zhang C (2012). Cardiac natriuretic peptides act via p38 MAPK to induce the brown fat thermogenic program in mouse and human adipocytes. J Clin Invest.

[69] Pan JA, Lin H, Yu JY, Zhang HL, Zhang JF, Wang CQ (2021). MiR-21-3p inhibits adipose browning by targeting FGFR1 and aggravates atrial fibrosis in diabetes. Oxid Med Cell Longev.

[70] Wu Y, Zhou S, Smas CM (2010). Downregulated expression of the secreted glycoprotein follistatin-like 1 (Fstl1) is a robust hallmark of preadipocyte to adipocyte conversion. Mech Dev.

[71] Napoli C, Schiano C, Soricelli A (2019). Increasing evidence of pathogenic role of the Mediator (MED) complex in the development of cardiovascular diseases. Biochimie.

[72] Souza SC, Chau MD, Yang Q, Gauthier MS, Clairmont KB, Wu Z (2011). Atrial natriuretic peptide regulates lipid mobilization and oxygen consumption in human adipocytes by activating AMPK. Biochem Biophys Res Commun.

[73] Zechner R, Zimmermann R, Eichmann TO, Kohlwein SD, Haemmerle G, Lass A (2012). FAT SIGNALS—lipases and lipolysis in lipid metabolism and signaling. Cell Metab.

[74] Cannone V, Cabassi A, Volpi R, Burnett JC. Atrial natriuretic peptide: a molecular target of novel therapeutic approaches to cardio-metabolic disease. Int J Mol Sci. 2019;20(13). 10.3390/ijms20133265 PMC6651335 31269783

[75] Sarzani R, Marcucci P, Salvi F, Bordicchia M, Espinosa E, Mucci L (2008). Angiotensin II stimulates and atrial natriuretic peptide inhibits human visceral adipocyte growth. Int J Obes (Lond).

[76] Grøntved L, Madsen MS, Boergesen M, Roeder RG, Mandrup S (2010). MED14 tethers mediator to the N-terminal domain of peroxisome proliferator-activated receptor gamma and is required for full transcriptional activity and adipogenesis. Mol Cell Biol.

[77] Wang W, Huang L, Huang Y, Yin JW, Berk AJ, Friedman JM (2009). Mediator MED23 links insulin signaling to the adipogenesis transcription cascade. Dev Cell.

[78] Suárez-Zamorano N, Fabbiano S, Chevalier C, Stojanović O, Colin DJ, Stevanović A (2015). Microbiota depletion promotes browning of white adipose tissue and reduces obesity. Nat Med.

[79] Fang D, Shi X, Lu T, Ruan H, Gao Y (2019). The glycoprotein follistatin-like 1 promotes brown adipose thermogenesis. Metabolism.

[80] Naruse K, Yamasaki Y, Tsunemi T, Onogi A, Noguchi T, Sado T (2011). Increase of high molecular weight adiponectin in hypertensive pregnancy was correlated with brain-type natriuretic peptide stimulation on adipocyte. Pregnancy Hypertens.

[81] Gruden G, Landi A, Bruno G (2014). Natriuretic peptides, heart, and adipose tissue: new findings and future developments for diabetes research. Diabetes Care.

[82] Tsukamoto O, Fujita M, Kato M, Yamazaki S, Asano Y, Ogai A (2009). Natriuretic peptides enhance the production of adiponectin in human adipocytes and in patients with chronic heart failure. J Am Coll Cardiol.

[83] Birkenfeld AL, Boschmann M, Engeli S, Moro C, Arafat AM, Luft FC (2012). Atrial natriuretic peptide and adiponectin interactions in man. PLoS ONE.

[84] D'Souza K, Nzirorera C, Kienesberger PC (2016). Lipid metabolism and signaling in cardiac lipotoxicity. Biochim Biophys Acta.

[85] Kimura H, Nagoshi T, Oi Y, Yoshii A, Tanaka Y, Takahashi H (2021). Treatment with atrial natriuretic peptide induces adipose tissue browning and exerts thermogenic actions in vivo. Sci Rep.

[86] Peterson JM, Wei Z, Seldin MM, Byerly MS, Aja S, Wong GW (2013). CTRP9 transgenic mice are protected from diet-induced obesity and metabolic dysfunction. Am J Physiol Regul Integr Comp Physiol.

[87] Song R, Peng W, Zhang Y, Lv F, Wu HK, Guo J (2013). Central role of E3 ubiquitin ligase MG53 in insulin resistance and metabolic disorders. Nature.

[88] Lee JH, Bassel-Duby R, Olson EN (2014). Heart- and muscle-derived signaling system dependent on MED13 and wingless controls obesity in drosophila. Proc Natl Acad Sci U S A.

[89] Fernandes T, Barretti DL, Phillips MI, Menezes OE (2018). Exercise training prevents obesity-associated disorders: role of miRNA-208a and MED13. Mol Cell Endocrinol.

[90] Carrer M, Liu N, Grueter CE, Williams AH, Frisard MI, Hulver MW (2012). Control of mitochondrial metabolism and systemic energy homeostasis by microRNAs 378 and 378*. Proc Natl Acad Sci U S A.

[91] Pol CJ, Pollak NM, Jurczak MJ, Zacharia E, Karagiannides I, Kyriazis ID (2019). Cardiac myocyte KLF5 regulates body weight via alteration of cardiac FGF21. Biochim Biophys Acta Mol Basis Dis.

[92] Blumensatt M, Fahlbusch P, Hilgers R, Bekaert M, de Wiza DH, Akhyari P (2017). Secretory products from epicardial adipose tissue from patients with type 2 diabetes impair mitochondrial β-oxidation in cardiomyocytes via activation of the cardiac renin-angiotensin system and induction of miR-208a. Basic Res Cardiol..

[93] Lahav R, Haim Y, Bhandarkar NS, Levin L, Chalifa-Caspi V, Sarver D (2021). CTRP6 rapidly responds to acute nutritional changes, regulating adipose tissue expansion and inflammation in mice. Am J Physiol Endocrinol Metab.

[94] Xu X, Zhang T, Mokou M, Li L, Li P, Song J (2020). Follistatin-like 1 as a novel adipomyokine related to insulin resistance and physical activity. J Clin Endocrinol Metab.

[95] Fan N, Sun H, Wang Y, Wang Y, Zhang L, Xia Z (2013). Follistatin-like 1: a potential mediator of inflammation in obesity. Mediators Inflamm.

[96] Horak M, Kuruczova D, Zlamal F, Tomandl J, Bienertova-Vasku J (2018). Follistatin-like 1 Is downregulated in morbidly and super obese central-european population. Dis Markers.

[97] Bae IS, Kim SH. Expression and secretion of an atrial natriuretic peptide in beige-like 3T3-L1 adipocytes. Int J Mol Sci. 2019;20(24). 10.3390/ijms20246128 PMC6940835 31817347

[98] Fiore D, Gianfrilli D, Giannetta E, Galea N, Panio G, di Dato C (2016). PDE5 inhibition ameliorates visceral adiposity targeting the miR-22/SIRT1 pathway: evidence from the CECSID trial. J Clin Endocrinol Metab.

[99] Ouchi N, Kihara S, Arita Y, Maeda K, Kuriyama H, Okamoto Y (1999). Novel modulator for endothelial adhesion molecules: adipocyte-derived plasma protein adiponectin. Circulation.

[100] Kumada M, Kihara S, Sumitsuji S, Kawamoto T, Matsumoto S, Ouchi N (2003). Association of hypoadiponectinemia with coronary artery disease in men. Arterioscler Thromb Vasc Biol.

[101] Kojima S, Funahashi T, Sakamoto T, Miyamoto S, Soejima H, Hokamaki J (2003). The variation of plasma concentrations of a novel, adipocyte derived protein, adiponectin, in patients with acute myocardial infarction. Heart.

[102] Du H, Li X, Zhao W, Jiang N (2022). The difference between sacubitril valsartan and valsartan on vascular endothelial function, APN, MMP-9, and BNP levels in patients with hypertension and chronic heart failure. J Healthc Eng.

[103] Djoussé L, Wilk JB, Hanson NQ, Glynn RJ, Tsai MY, Gaziano JM (2013). Association between adiponectin and heart failure risk in the physicians' health study. Obesity.

[104] Sunaga H, Koitabashi N, Iso T, Matsui H, Obokata M, Kawakami R (2019). Activation of cardiac AMPK-FGF21 feed-forward loop in acute myocardial infarction: role of adrenergic overdrive and lipolysis byproducts. Sci Rep.

[105] Brahma MK, Adam RC, Pollak NM, Jaeger D, Zierler KA, Pöcher N (2014). Fibroblast growth factor 21 is induced upon cardiac stress and alters cardiac lipid homeostasis. J Lipid Res.

[106] Sommakia S, Almaw NH, Lee SH, Ramadurai DKA, Taleb I, Kyriakopoulos CP (2022). FGF21 (fibroblast growth factor 21) defines a potential cardiohepatic signaling circuit in end-stage heart failure. Circ Heart Fail.

[107] Grueter CE, van Rooij E, Johnson BA, DeLeon SM, Sutherland LB, Qi X (2012). A cardiac microRNA governs systemic energy homeostasis by regulation of MED13. Cell.

[108] Becker-Greene D, Li H, Perez-Cremades D, Wu W, Bestepe F, Ozdemir D (2021). MiR-409-3p targets a MAP4K3-ZEB1-PLGF signaling axis and controls brown adipose tissue angiogenesis and insulin resistance. Cell Mol Life Sci.

[109] Montaigne D, Butruille L, Staels B (2021). PPAR control of metabolism and cardiovascular functions. Nat Rev Cardiol.

[110] Fang D, Shi X, Jia X, Yang C, Wang L, Du B (2022). Ups and downs: The PPARγ/p-PPARγ seesaw of follistatin-like 1 and integrin receptor signaling in adipogenesis. Mol Metab.

[111] Song E, Da Eira D, Jani S, Sepa-Kishi D, Vu V, Hunter H (2021). Cardiac autophagy deficiency attenuates anp production and disrupts myocardial-adipose cross talk, leading to increased fat accumulation and metabolic dysfunction. Diabetes.

[112] Liu D, Ceddia RP, Collins S (2018). Cardiac natriuretic peptides promote adipose ‘browning’ through mTOR complex-1. Mol Metab.

[113] Bordicchia M, Spannella F, Ferretti G, Bacchetti T, Vignini A, Di Pentima C (2019). PCSK9 is expressed in human visceral adipose tissue and regulated by insulin and cardiac natriuretic peptides. Int J Mol Sci..

[114] Han C, Yang J, Zhang E, Jiang Y, Qiao A, Du Y (2022). Metabolic labeling of cardiomyocyte-derived small extracellular-vesicle (sEV) miRNAs identifies miR-208a in cardiac regulation of lung gene expression. J Extracell Vesicles.

[115] Birkenfeld AL, Budziarek P, Boschmann M, Moro C, Adams F, Franke G (2008). Atrial natriuretic peptide induces postprandial lipid oxidation in humans. Diabetes.

[116] Polak J, Kotrc M, Wedellova Z, Jabor A, Malek I, Kautzner J (2011). Lipolytic effects of B-type natriuretic peptide 1–32 in adipose tissue of heart failure patients compared with healthy controls. J Am Coll Cardiol.

[117] Gentili A, Frangione MR, Albini E, Vacca C, Ricci MA, De Vuono S (2017). Modulation of natriuretic peptide receptors in human adipose tissue: molecular mechanisms behind the “natriuretic handicap” in morbidly obese patients. Transl Res.

[118] Wang L, Tang Y, Herman MA, Spurney RF (2023). Pharmacologic blockade of the natriuretic peptide clearance receptor promotes weight loss and enhances insulin sensitivity in type 2 diabetes. Transl Res.

[119] Han F, Zhang Y, Shao M, Mu Q, Jiao X, Hou N (2018). C1q/TNF-related protein 9 improves the anti-contractile effects of perivascular adipose tissue via the AMPK-eNOS pathway in diet-induced obese mice. Clin Exp Pharmacol Physiol.

[120] Abumrad NA, Cabodevilla AG, Samovski D, Pietka T, Basu D, Goldberg IJ (2021). Endothelial cell receptors in tissue lipid uptake and metabolism. Circ Res.

[121] Soppert J, Lehrke M, Marx N, Jankowski J, Noels H (2020). Lipoproteins and lipids in cardiovascular disease: from mechanistic insights to therapeutic targeting. Adv Drug Deliv Rev.

